# Development of Thermo- and pH-Sensitive Liposomal Magnetic Carriers for New Potential Antitumor Thienopyridine Derivatives

**DOI:** 10.3390/ma15051737

**Published:** 2022-02-25

**Authors:** Beatriz C. Ribeiro, Cristina A. R. Alvarez, Bárbara C. Alves, Juliana M. Rodrigues, Maria João R. P. Queiroz, Bernardo G. Almeida, Ana Pires, André M. Pereira, João P. Araújo, Paulo J. G. Coutinho, Ana Rita O. Rodrigues, Elisabete M. S. Castanheira

**Affiliations:** 1 Centre of Physics of Minho and Porto Universities (CF-UM-UP), Campus de Gualtar, University of Minho, 4710-057 Braga, Portugal; pg37976@alunos.uminho.pt (B.C.R.); pg46653@alunos.uminho.pt (C.A.R.A.); pg46652@alunos.uminho.pt (B.C.A.); bernardo@fisica.uminho.pt (B.G.A.); pcoutinho@fisica.uminho.pt (P.J.G.C.); 2 Centre of Chemistry (CQUM), University of Minho, Campus de Gualtar, 4710-057 Braga, Portugal; juliana.mourarodrigues@gmail.com (J.M.R.); mjrpq@quimica.uminho.pt (M.J.R.P.Q.); 3 IFIMUP-Instituto de Física dos Materiais, Universidade do Porto, Rua do Campo Alegre, 4169-007 Porto, Portugal; ana.pires@fc.up.pt (A.P.); ampereira@fc.up.pt (A.M.P.); jearaujo@fc.up.pt (J.P.A.)

**Keywords:** magnetic nanoparticles, mixed ferrite, magnetoliposomes, pH sensitive, thienopyridine derivatives, antitumor compounds, cancer therapy

## Abstract

The development of stimuli-sensitive drug delivery systems is a very attractive area of current research in cancer therapy. The deep knowledge on the microenvironment of tumors has supported the progress of nanosystems’ ability for controlled and local fusion as well as drug release. Temperature and pH are two of the most promising triggers in the development of sensitive formulations to improve the efficacy of anticancer agents. Herein, magnetic liposomes with fusogenic sensitivity to pH and temperature were developed aiming at dual cancer therapy (by chemotherapy and magnetic hyperthermia). Magnetic nanoparticles of mixed calcium/manganese ferrite were synthesized by co-precipitation with citrate and by sol–gel method, and characterized by X-ray diffraction (XRD), scanning electron microscopy in transmission mode (STEM), and superconducting quantum interference device (SQUID). The citrate-stabilized nanoparticles showed a small-sized population (around 8 nm, determined by XRD) and suitable magnetic properties, with a low coercivity and high saturation magnetization (~54 emu/g). The nanoparticles were incorporated into liposomes of dipalmitoylphosphatidylcholine/cholesteryl hemisuccinate (DPPC:CHEMS) and of the same components with a PEGylated lipid (DPPC:CHEMS:DSPE-PEG), resulting in magnetoliposomes with sizes around 100 nm. Dynamic light scattering (DLS) and electrophoretic light scattering (ELS) measurements were performed to investigate the pH-sensitivity of the magnetoliposomes’ fusogenic ability. Two new antitumor thienopyridine derivatives were efficiently encapsulated in the magnetic liposomes and the drug delivery capability of the loaded nanosystems was evaluated, under different pH and temperature conditions.

## 1. Introduction

Tumors are characterized by a specific microenvironment due to the uncontrolled cell proliferation, acidic pH, overexpression of proteins and enzymes and high levels of oxidation/deoxidation, as a result of the peculiar nutritional environment and of the metabolic pattern change of tissues. Based on these characteristics, endogenous and exogenous stimuli have been strategically studied as triggers in the development of controlled drug-delivery nanosystems [1,2]. Endogenous triggers are regulated by the diseased tissue microenvironment, while exogenous ones can be modulated by external factors, and thus precisely controlled. Nanosystems with responsive profiles to the pH, redox, enzyme and ionic microenvironment have been the most reported endogenous triggers. On the other hand, the most used exogenous triggers are temperature, magnetic field, light, electric field and ultrasound [3]. Magnetic fields can precisely control heat generation to induce drug release from thermo-responsive nanosystems, while overheating cancer cells [4,5]. Under AC magnetic field, superparamagnetic nanoparticles are able to produce heat, and nanosystems based on magnetic nanoparticles are ideal for temperature-controlled drug delivery and simultaneous hyperthermia. Metal ferrite nanoparticles are promising for their high saturation magnetization, low hysteresis and chemical stability. Among all ferrites, manganese ferrite nanoparticles have been reported as novel agents for magnetic hyperthermia for their tunable magnetic properties [6]. The magnetic properties of metal ferrites with spinel structure strongly depend on the nature of the ions and their distribution among tetrahedral and octahedral sites. Hence, doping spinel ferrites with non-toxic and non-magnetic elements, such as calcium, alters the distribution of ions in both sites, leading to variation in magnetic properties that can boost magnetization, while promoting a higher biocompatibility [7,8].

The integration of two or more stimuli (endogenous and/or exogenous) into a single nanosystem is very attractive and has been explored to enhance therapeutic efficacy. Multi-responsive drug-delivery nanosystems with different stimuli combinations have been reported [9,10,11]. Considering the acidic pH of tumors, with the extracellular environment reaching pH values down to 5.7 [12], and the fact that high temperatures can trigger drug release in thermo-sensitive nanosystems, while selectively damaging cancer cells (in the range of 39 °C to 42 °C) [13], the combination of pH and temperature stimuli is a promising approach in cancer treatment. The potential of cholesteryl hemisuccinate (CHEMS) to achieve nanosystems with pH-dependent drug delivery has been investigated. CHEMS is a protonable lipid that is negatively charged at neutral pH and becomes neutral at acidic environment. This weakly acidic amphiphilic molecule possesses polymorphic phase behavior as a function of its protonation state around its pK_a_, owning a lamellar stable phase at pH = 7 and an inverted hexagonal phase (H_II_) at pH = 5 [14]. Hence, in an acidic environment, formulations containing CHEMS become fusogenic. In fact, lipids that undergo the lamellar-to-inverted hexagonal phase support membrane fusion, while phospholipids that undergo the gel-to-liquid crystalline phase promote drug release due to the increased bilayer fluidity and permeability above melting temperature. For thermosensitive formulations, DPPC is one of the most suitable lipids because it has a transition temperature around 41 °C, that is, a few degrees above physiological temperature and in the range of mild hyperthermia temperatures [4]. The effect of CHEMS as a stabilizer of DPPC vesicles shows that the inclusion of a low amount of CHEMS has no significant influence on the transition temperature of the formulation [15].

Hence, multi-stimuli magnetoliposomes of DPPC and CHEMS can be very attractive, due to their ability to be stable in blood circulation and release cargo at target sites by internal (pH) and external (magnetic field) stimuli while performing hyperthermia, which is promising for cancer treatment.

**Figure 1:**
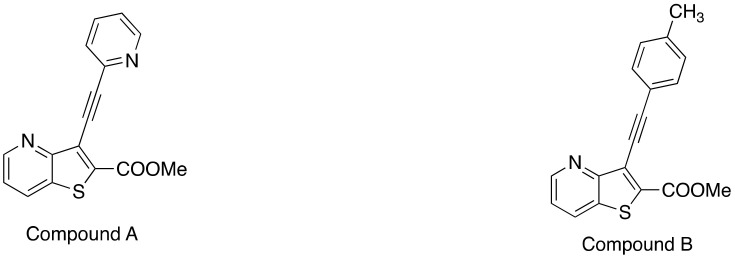
Structures of the new antitumor compounds (Me: methyl group). Compound A: methyl 3-(pyridin-2-ylethynyl)thieno[3,2-*b*]pyridine-2-carboxylate; Compound B: methyl 3-(p-tolyl-ethynyl)thieno[3,2-*b*]pyridine-2-carboxylate.

In this work, magnetoliposomes based on the thermosensitive lipid DPPC and the pH-sensitive agent CHEMS were prepared. Magnetic nanoparticles of manganese/calcium ferrite were chosen as the magnetic component. To assess the potential of the developed magnetic liposomes as drug nanocarriers, two novel antitumor thienopyridine derivatives, recently synthesized [16] (Figure 1), were loaded into the nanosystems. The thienopyridine derivatives have been described as antitumor and antiangiogenic agents, as well as inhibitors for tyrosine kinase receptors [17,18,19]. 

The two novel compounds were previously assayed in human tumor cell lines [16] (Table 1), namely in colon cancer (HCT-15 cell line) and non-small cell lung cancer (NCI-H460). The results were compared with those for doxorubicin (DOX), a widely-used therapeutic anticancer agent, and revealed much lower growth inhibitory concentrations (GI_50_) for the new compounds in both tumor cell lines (except for Compound A in NCI-H460 cells). These GI_50_ values, in the nanomolar concentration range, stand out when compared to other thienopyridines [18,19,20,21,22] and to the anticancer DOX, inspiring the assays of encapsulation in magnetoliposomes.

Both compounds were successfully encapsulated in the developed nanosystems, representing promising formulations for application in cancer therapeutics.

## 2. Materials and Methods

### 2.1. Preparation of Mixed Calcium/Manganese Ferrite Nanoparticles

Spectroscopic-grade solvents and ultrapure water of Milli-Q grade (MilliporeSigma, St. Louis, MO, USA) were used in all preparations. Iron(III) chloride (FeCl_3_·6H_2_O,), manganese sulfate (MnSO_4_·H_2_O) and calcium acetate, Ca(CH_3_COO)_2_·H_2_O (from Sigma-Aldrich, St. Louis, MO, USA) were used in the synthesis of the mixed calcium/manganese ferrite nanoparticles. ACS-grade reagent trisodium citrate dehydrate and sodium hydroxide solution, 50% in water (from Sigma-Aldrich, St. Louis, MO, USA), were employed in the co-precipitation method. Nitric acid and citric acid (from Sigma-Aldrich, St. Louis, MO, USA) were used in the sol–gel synthesis method.

#### 2.1.1. Co-Precipitation Method

Citrate-stabilized calcium/manganese ferrite NPs, Ca_0.5_Mn_0.5_Fe_2_O_4_, were prepared by the co-precipitation method in aqueous solution. First, an aqueous solution of NaOH (2.32 M) and trisodium citrate dehydrate (0.75 M) was prepared and heated up to 90 °C, under vortexing. Then, a mixed solution containing iron(III) chloride 1 M, manganese sulfate 0.25 M and calcium acetate 0.25 M was added drop-by-drop. The mixture was kept under vortexing, at 90 °C, for 2 h. The obtained nanoparticles were washed several times by magnetic decantation with water and ethanol.

#### 2.1.2. Sol-Gel Method

The Ca_0.5_Mn_0.5_Fe_2_O_4_ nanoparticles were also synthesized via the sol–gel method under acid-catalyzed conditions. Nitric acid 0.05 M and citric acid 0.15 M were used for the hydrolysis of the metal precursor mixture. A mixed solution of iron(III) chloride 1 M, manganese sulfate 0.25 M and calcium acetate 0.25 M, was added to the acids and slowly heated at 90 °C, under magnetic stirring, to form a xerogel. After formation of the dry gel, the temperature was raised to 250 °C, until a powder was obtained. The purification process was performed by several cycles of centrifugation, washing with water and ethanol. Finally, the obtained nanoparticles were subjected to calcination at 300 °C for 3 h.

### 2.2. Synthesis of Magnetic Liposomes and Small Unilamellar Vesicles

Magnetic liposomes based on Ca_0.5_Mn_0.5_Fe_2_O_4_ nanoparticles were obtained by the ethanolic injection method, as previously described [23,24]. Briefly, an ethanolic solution of 1 × 10^−3^ M total lipid concentration was injected, drop by drop, into a nanoparticle aqueous solution (1 × 10^−4^ M) at 55 °C under vortexing. The non-encapsulated nanoparticles were removed through magnetic decantation. Different formulations containing the thermosensitive lipid DPPC (dipalmitoylphosphatidylcholine), the pH-sensitive agent cholesteryl hemisuccinate (CHEMS) and the PEGylated lipid distearoylphosphatidylethanolamine-*N*-[methoxy(polyethylene glycol)-2000] (ammonium salt) (DSPE-PEG) were prepared, specifically DPPC (100%), DPPC:CHEMS (molar ratio 80:20) and DPPC:CHEMS:DSPE-PEG (molar ratio 80:20:0.4). All components were purchased from Sigma-Aldrich (St. Louis, MO, USA).

The two novel antitumor thienopyridine derivatives were encapsulated into the magnetoliposomes by co-injection of the compound with the lipid solution (final compound concentration: 1 × 10^−6^ M), an efficient method for encapsulation of hydrophobic compounds [25].

Small unilamellar vesicles (SUVs) were used as models of cell membranes, and their interaction with the developed magnetoliposomes was studied. SUVs of egg-phosphatidylcholine, Egg-PC (from Sigma-Aldrich, St. Louis, MO, USA) were also prepared by ethanolic injection method.

### 2.3. Structural Characterization

The composition and crystalline phases of the synthesized nanoparticles were evaluated by the XRD (X-Ray Diffraction) technique, using a PAN’alytical X’Pert PRO diffractometer, operating with CuK_α_ radiation and Bragg–Brentano configuration, at the Electron Microscopy Unit, University of Trás-os-Montes and Alto Douro (UTAD), Vila Real, Portugal.

Scanning electron microscopy images were recorded using a NanoSEM-FEI Nova 200 (FEI Technologies, Inc., Hillsboro, OR, USA), operating in transmission mode (STEM), at SEMAT (Serviços de Caracterização de Materiais, Guimarães, Portugal). The software *ImageJ* (National Institutes of Health (NIH), version 1.53c, Bethesda, MD, USA) was used to process STEM images by increasing contrast and subtracting background. Then, a manual outline of the nanoparticles with the best-defined limit was performed (~150 counts) using the ROI (Region of Interest) manager tool, and the sizes were estimated considering the area of the circle.

The size (hydrodynamic diameter) and zeta potential of magnetic liposomes were measured using a Dynamic Light Scattering NANO ZS Malvern Zetasizer (Malvern Panalytical Ltd., Malvern, UK) equipped with a He-Ne laser (λ = 632.8 nm). For each sample, five independent measurements were carried out, to determine mean size and size distribution (polydispersity index).

### 2.4. Magnetic Measurements

The magnetic properties of the Ca_0.5_Mn_0.5_Fe_2_O_4_ nanoparticles were measured in a MPMS3 SQUID magnetometer MPMS5XL (Quantum Design Inc., San Diego, CA, USA), using applied magnetic fields up to 5 T. The magnetization dependence on magnetic field (hysteresis cycles) was performed by measuring the magnetization at a series of different applied magnetic fields, at room temperature.

### 2.5. Spectroscopic Measurements

#### 2.5.1. General Methods

The UV-Vis-NIR spectrophotometer Shimadzu UV-3600 Plus (Shimadzu Corporation, Kyoto, Japan) was used to measure the absorption spectra and the spectrofluorimeter Fluorolog 3 (HORIBA Jobin Yvon IBH Ltd., Glasgow, UK), possessing double monochromators in excitation and emission, Glan–Thompson polarizers, and a temperature-controlled sample holder, was used to measure the emission spectra.

#### 2.5.2. Fluorescence Anisotropy Measurements

Fluorescence anisotropy studies were performed by measuring the steady-state fluorescence anisotropy (*r*) [26], calculated by Equation (1)

(1) $$r=\frac{{\;I}_{\mathrm{VV}}-{\mathrm{GI}}_{\mathrm{VH}}}{I_{\mathrm{VV}}+2{\mathrm{GI}}_{\mathrm{VH}}\;},$$

where I_VV_ and I_VH_ are the intensities of the emission spectra obtained with vertical and horizontal polarization, respectively (using excitation light with vertical polarization). The instrument correction factor (*G*) is the ratio I_HV_/I_HH_, where I_HV_ and I_HH_ are the emission intensities obtained with vertical and horizontal polarization (using excitation light with horizontal polarization).

#### 2.5.3. Compound Encapsulation Efficiency

The encapsulation efficiency, EE(%), of the antitumor compounds in the magnetic nanosystems was determined by using Amicon^®^ Ultra centrifugal filter units 100 kDa (Merck Millipore, Darmstadt, Germany) for the separation of encapsulated and non-encapsulated compound. For that, drug-loaded magnetoliposomes were subjected to a 60 min centrifugation at 11,000 rpm and the filtrate (consisting of non-encapsulated drug) fluorescence was measured. Then, the concentration of non-encapsulated compound was determined through a previously obtained calibration curve for each compound (plot of the fluorescence intensity versus compound concentration). For that, the fluorescence intensity was measured and converted to the corresponding compound concentration. For each lipid formulation, three independent measurements were carried out and EE(%) was determined using Equation (2).

(2) $$\mathrm{EE}\left(\%\right)=\frac{C_{\left(\mathrm{total}\;\mathrm{compound}\right)}-{\;C}_{\left(\mathrm{non}-\mathrm{encapsulated}\;\mathrm{compound}\right)}}{C_{\left(\mathrm{total}\;\mathrm{compound}\right)}}\times 100$$

## 3. Results and Discussion

### 3.1. Nanoparticle Synthesis and Characterization

#### 3.1.1. X-ray Analysis

**Figure 2:**
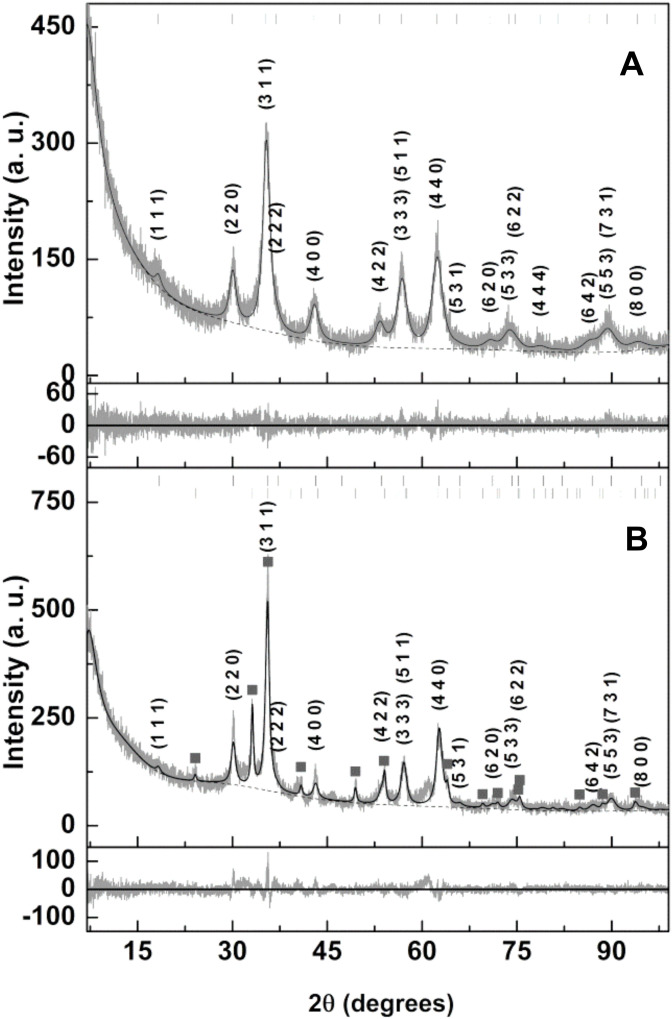
X-Ray diffractogram of (**A**) citrate-stabilized calcium/manganese ferrite nanoparticles; (**B**) nanoparticles prepared by sol–gel method.

Ca_0.5_Mn_0.5_Fe_2_O_4_ NPs were prepared by the co-precipitation method in the presence of citrate (to provide electrostatic stabilization) and by the sol–gel technique. The X-ray diffraction (XRD) pattern of the synthesized nanoparticles was obtained and the data analysis was processed by Rietveld optimization using the Profex/BGMN software [27,28] to identify the sample phases and crystallite sizes. The XRD pattern of the Ca_0.5_Mn_0.5_Fe_2_O_4_ (citrate-stabilized) NPs displayed in Figure 2A shows well-defined peaks, confirming its crystallinity. The needed structure file resulted from the import process of CIF file nr. 2300618 (MnFe_2_O_4_, space group Fd-3m) followed by changes in the unit cell composition, so that half the Mn^2+^ positions are occupied by Ca^2+^ while the cation distribution over the tetrahedral and octahedral sites can be varied during the Rietveld optimization. All the identified peaks correspond to the intended phase, confirming its purity, and occurred at 18.2° (1 1 1), 29.9° (2 2 0), 35.3° (3 1 1), 36.9° (2 2 2), 42.9° (4 0 0), 46.9° (3 3 1), 53.2° (4 2 2), 56.7° (5 1 1), 56.7° (3 3 3), 62.3° (4 4 0), 65.5° (5 3 1), 70.6° (6 2 0), 73.7° (5 3 3), 74.7° (6 2 2), 78.6° (4 4 4), 81.5° (7 1 1), 81.5° (5 5 1), 86.3° (6 4 2), 89.2° (7 3 1), 89.2° (5 5 3), 94.0° (8 0 0) and 96.9° (7 3 3). On the other hand, the corresponding XRD of the Ca_0.5_Mn_0.5_Fe_2_O_4_ (sol–gel) NPs (Figure 2B) revealed additional peaks indicating that the sol–gel method results in less pure samples. Almost all of the additional peaks observed match with the hematite phase (Hematite.str included in BGMN structure files), as observed by the identified peaks (filled squares). A percentage of 27.3% of hematite was detected. The success of the sol–gel method relies on the uniform distribution of metal cations within the xerogel, prior to the high-temperature combustion process. The fact that a significant amount of hematite was obtained indicates the presence of iron-enriched regions in the prepared xerogel.

The crystallite sizes were estimated by the peak broadening effect as implemented in BGMN. The results of Rietveld optimization are indicated in Table 2 and in Figure 2. Sizes of 7.9 nm and 10.2 nm were obtained for the Ca_0.5_Mn_0.5_Fe_2_O_4_ (citrate-stabilized) and Ca_0.5_Mn_0.5_Fe_2_O_4_ (sol–gel) NPs, respectively. For the case of the citrate-stabilized NPs, better fits were obtained for cation distributions that placed Ca^2+^ in octahedral sites. The obtained lattice parameter of 0.8427 nm is larger than the value of 0.8387 reported for MnFe_2_O_4_ produced by a hydrothermal procedure [29]. This shows that an expansion of the crystal structure occurs in order to accommodate the Ca^2+^ ions.

#### 3.1.2. Scanning Electron Microscopy (SEM)

**Figure 3:**
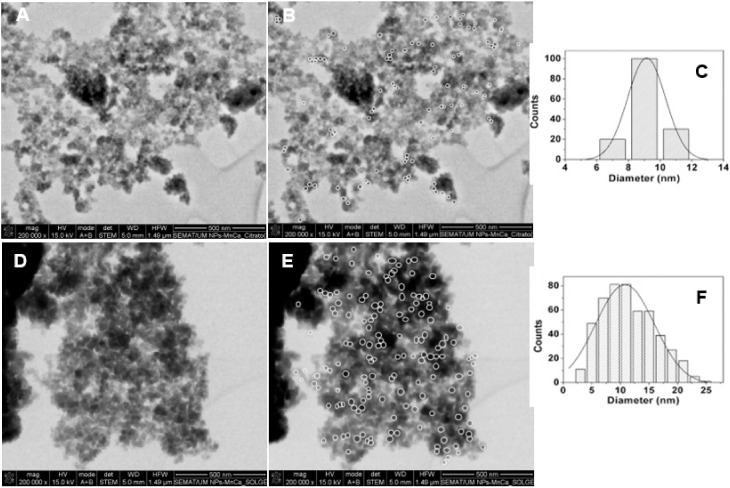
STEM images of (**A**) citrate-stabilized nanoparticles and (**B**) citrate-stabilized nanoparticles with the nanoparticles selected by *Image J* software (white circles); (**C**) Histogram of size distribution of citrate-stabilized nanoparticles. STEM images of (**D**) nanoparticles prepared by sol–gel and (**E**) nanoparticles prepared by sol–gel with the nanoparticles selected by *Image J* software (white circles); (**F**) Histogram of size distribution of nanoparticles prepared by sol–gel.

The SEM images of Ca_0.5_Mn_0.5_Fe_2_O_4_ nanoparticles are presented in Figure 3. A Gaussian distribution was fitted to the experimental data and populations of 9.1 ± 2.4 nm and 11.5 ± 4.3 nm were obtained, respectively, for the citrate-stabilized NPs (Figure 3A) and NPs obtained by sol–gel method (Figure 3B). These size values are in very good agreement with XRD results. The small and uniform population of the citrate-stabilized NPs, with low size distributions, emphasizes the role of citrate, which demonstrated an important role in the synthesis of ferrite nanoparticles, allowing for a homogeneous mixing of metal cations while retarding particle growth via the formation of surface citrate complexes, inhibiting the agglomeration of the NPs [30,31]. Some particle aggregation observed in the images is due to the technique employed, which requires a dry film of the sample in a solid grid with subsequent application of a vacuum. This procedure causes the aggregation of the nanostructures in the grid and, thus, in SEM images.

#### 3.1.3. Sedimentation Kinetics

**Figure 4:**
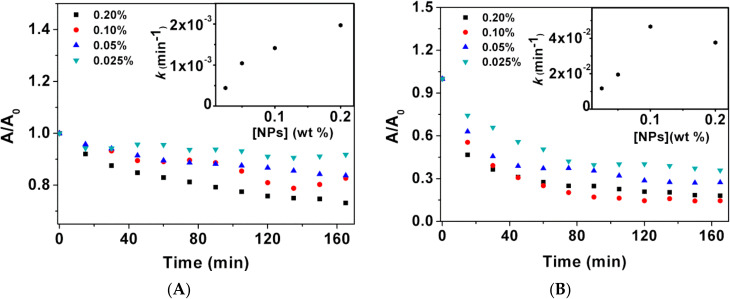
Sedimentation profiles of (**A**) citrate-stabilized nanoparticles; (**B**) Nanoparticles prepared by sol–gel. Insets: sedimentation rate dependence on nanoparticle concentration.

The colloidal stability of the nanoparticles is an important parameter for biomedical applications. Hence, the sedimentation profile of suspensions of the prepared nanoparticles is crucial in determining their stability. Different concentrations of the prepared nanoparticles, 0.2%, 0.1%, 0.05% and 0.025% (% m/v), were studied. The deposition rate was determined through the sedimentation kinetics, which was obtained measuring the absorbance of nanoparticles suspensions within 15 min intervals, for 3 h (Figure 4). The experimental results follow first-order kinetics and the sedimentation rates were estimated by fitting the Becquerel decay function or compressed hyperbola to the sedimentation profiles at different concentrations (insets of Figure 4). Becquerel’s decay law is given by Equation (3),

(3) It=11+ctτ01/c

where the control parameter c is taken as 0 < c < 1, and $\tau_{0}$ has dimensions of time [32].

Both types of nanoparticles (synthesized by the two different methods) were shown to be stable, with sedimentation behavior suggesting the occurrence of nanoparticle aggregation into stable agglomerates which settle down at a faster rate than single nanoparticles [33]. Within the range of concentrations studied, the deposition of the citrate-stabilized NPs shows a linear trend with increasing concentration. On the other hand, the NPs prepared by sol–gel revealed a faster decay over time, with no linear dependence with the NP concentration. The citrate-stabilized NPs display higher stability for the largest concentration studied, with a deposition rate of 1.98 × 10^−3^ min^−1^ for 0.2 wt%, while sol–gel NPs show a rate of 3.94 × 10^−2^ min^−1^ for the same nanoparticle concentration. The three carboxyl groups in every citrate ion and the repulsive forces between the electric charges of the radical ions make the nanoparticles more water-dispersible, providing electrostatic stabilization [34].

#### 3.1.4. Magnetic Properties

**Figure 5:**
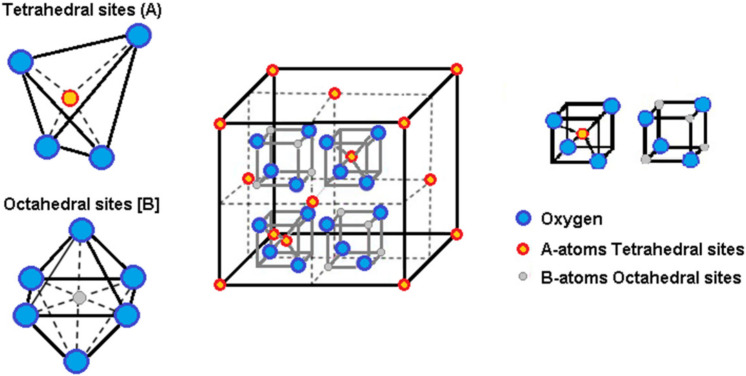
Representation of the crystal cell lattice, with the representation of the A-sites and B-sites.

Ferrite nanoparticles typically have a spinel-type crystal structure with a general formula (A)[B]_2_O_4_, where (A) denotes tetrahedral sites and [B] the octahedral sites. Each unit cell is composed of eight formula units, where the larger oxygen anions enclose a face-centered-cubic structure with the smaller cations in the interstitial sites, that is, (A)-sites and [B]-sites (Figure 5). The cation distribution in the (A)-sites and [B]-sites, the magnetic interaction between the magnetic moments of the metal ions, and their relative ion strength determine the magnetic behavior of the nanoparticles.

The spinel structure of the mixed calcium/manganese ferrite can be written as $\left({\mathrm{Ca}}_{x}^{2+}{\mathrm{Mn}}_{y}^{2+}{\mathrm{Fe}}_{1-x-y}^{3+}\right)\;\left[{\mathrm{Ca}}_{0.5-x}^{2+}{\mathrm{Mn}}_{0.5-y}^{2+}{\mathrm{Fe}}_{1+x+y}^{3+}\right]O_{4}^{2-}$, where (1*-x-y*) denotes the inversion degree corresponding to the fraction of (A)-sites that are occupied by Fe^3+^ [35]. In an inverted spinel ferrite, one-half of Fe^3+^ is placed in (A)-sites and the other half in [B]-sites, mutually compensating their magnetic moments. Thus, the resulting magnetic moment of the ferrite is due to the magnetic moment of bivalent cations (Me^2+^) in [B]-sites [36]. According to XRD analysis, an octahedral site preference of Ca^2+^ was observed, from either a completely inverted structure or a structure with an inversion degree of 0.5 and Mn^2+^ only in (A)-sites (Table 3). Hence, the spinel structure takes either the formula $\left({\mathrm{Fe}}^{3+}\right)\left[{\mathrm{Ca}}_{0.5}^{2+}{\mathrm{Fe}}^{3+}\right]O_{4}^{2-}$ or $\left({\mathrm{Mn}}_{0.5}^{2+}{\mathrm{Fe}}_{0.5}^{3+}\right)\;\left[{\mathrm{Ca}}_{0.5}^{2+}{\mathrm{Fe}}_{1.5}^{3+}\right]{\;O}_{4}^{2-}.$ Bulk MnFe_2_O_4_ is a partially inverted spinel structure, with a typical small inversion parameter of 0.2 and a corresponding formula $\left({\mathrm{Mn}}_{0.8}^{2+}{\mathrm{Fe}}_{0.2}^{3+}\right)\;\left[{\mathrm{Mn}}_{0.2}^{2+}{\mathrm{Fe}}_{1.8}^{3+}\right]{\;O}_{4}^{2-}$. This is originated from the similar site preferences of Mn^2+^ and Fe^3+^, as both have similar sizes and d-orbital energy and occupation (d^5^) [37]. Therefore, the most probable configuration for the synthesized Ca_0.5_Mn_0.5_Fe_2_O_4_ nanoparticles is $\left({\mathrm{Mn}}_{0.5}^{2+}{\mathrm{Fe}}_{0.5}^{3+}\right)\;\left[{\mathrm{Ca}}_{0.5}^{2+}{\mathrm{Fe}}_{1.5}^{3+}\right]{\;O}_{4}^{2-}$.

**Figure 6:**
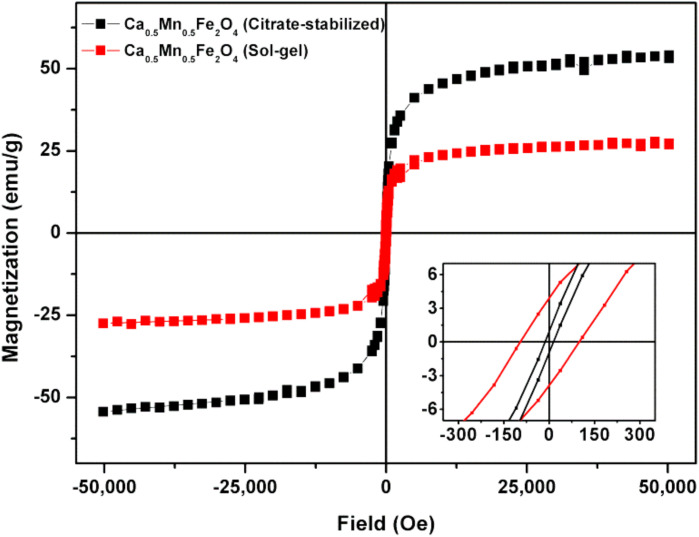
Hysteresis loops of citrate-stabilized Ca_0.5_Mn_0.5_Fe_2_O_4_ NPs and Ca_0.5_Mn_0.5_Fe_2_O_4_ NPs prepared by sol–gel, at room temperature. Inset: Low region field enlargement.

The magnetic dependence on the applied magnetic field of the citrate-stabilized Ca_0.5_Mn_0.5_Fe_2_O_4_ NPs and those prepared by sol–gel was measured and the corresponding hysteresis loops are shown on Figure 6. The obtained saturation magnetization and hysteresis parameters are summarized in Table 3.

Both types of Ca_0.5_Mn_0.5_Fe_2_O_4_ nanoparticles show no hysteresis, with very low values of remnant magnetization and coercive field (Table 3). The citrate-stabilized NPs are superparamagnetic, with a magnetic squareness value below 0.1, indicating the loss of more than 90% of magnetization upon the removal of the applied field. On the other hand, NPs obtained by sol–gel are at the limit for ferromagnetic behavior. A high saturation magnetization of 53.91 emu/g was obtained for the crystalline Ca_0.5_Mn_0.5_Fe_2_O_4_ citrate-stabilized nanoparticles, while poor magnetization was observed for the NPs obtained by sol–gel. The lower magnetization value of the nanoparticles synthesized by the sol–gel method can be justified by the presence of an additional phase of hematite (as detected in XRD diffractogram) which is very weakly magnetic.

According to Neel’s sub-lattice field model [7], the A-B exchange interaction is stronger than the A-A or B-B interaction and the saturation magnetization (M_s_) can be estimated by the relation M_s_ = M_B_ − M_A_, where M_B_ and M_A_ are the magnetization of [B]- and (A)-sites, respectively. Taking the magnetic moment of 5µ_B_ for the cations Mn^2+^ e Fe^3+^ (both with five unpaired d-electrons) and 0µ_B_ for Ca^2+^, the magnetic moment per formula unit of the $\left({\mathrm{Mn}}_{0.5}^{2+}{\mathrm{Fe}}_{0.5}^{3+}\right)\;\left[{\mathrm{Ca}}_{0.5}^{2+}{\mathrm{Fe}}_{1.5}^{3+}\right]{\;O}_{4}^{2-}$ configuration is given by [(Ca^2+^↑ 0.5 × 0µ_B_) + (Fe^3+^↑ 1.5 × 5µ_B_)] − ((Mn^2+^↓ 0.5 × 5µ_B_)+(Fe^3+^↓ 0.5 × 5µ_B_)] = 2.5µ_B_. On the other hand, assuming the typically small inversion parameter of manganese ferrite nanoparticles (*i* = 0.2), the expected magnetic moment per formula unit is [(Mn^2+^ ↑ 0.2 × 5µ_B_) + (Fe^3+^↑ 1.8 × 5µ_B_)] − ((Mn^2+^↓ 0.8 × 5µ_B_) + (Fe^3+^↓ 0.2 × 5µ_B_)] = 5µ_B_. Hence, a reduction in the magnetization for the Ca_0.5_Mn_0.5_Fe_2_O_4_ nanoparticles should be expected by the Neel’s sub-lattice field model. Yet, Wang et al. have prepared MnFe_2_O_4_ NPs with a saturation magnetization of 53.6 emu/g, using a synthesis process that resulted in nanoparticles with comparable crystalline sizes around 11.4 nm and a lattice parameter of 0.8387 nm [29]. Contrary to expectations, the magnetization reported by Wang for MnFe_2_O_4_ NPs is very similar to that obtained in this work for the citrate-stabilized Ca_0.5_Mn_0.5_Fe_2_O_4_ NPs (53.91 emu/g, Table 3). In addition, the increase in the lattice parameter from 0.8387 nm (MnFe_2_O_4_ NPs) to 0.8427 nm (Ca_0.5_Mn_0.5_Fe_2_O_4_, Table 2) points to a structural distortion that could disrupt the perfect alignment of spins from the A and B sites, leading to a possible increase in the effective magnetic moment. The lattice parameter increases and the Ca^2+^ octahedral site preference is supported by the larger ionic radius of the cation Ca^2+^ (0.99 Å contrasting with 0.80 Å of the Mn^2+^, and 0.64 Å of the Fe^3+^, for coordination 6 in octahedral position [38]). The influence of structural distortions in effective magnetic moment was also suggested by Y. Wang et al. for the case of rare-earth-doped calcium manganite perovskites [39]. Hence, the high magnetization of mixed calcium/manganese ferrite NPs could be related to the distortion that augments the magnetic moment of the system.

### 3.2. Magnetoliposome Characterization by DLS and SEM

Considering the best structural and magnetic properties of the citrate-stabilized nanoparticles obtained by co-precipitation, these NPs were chosen for the preparation of magnetic liposomes. The thermosensitive lipid DPPC (dipalmitoylphosphatidylcholine), possessing a transition (melting) temperature at 41 °C [40], around the ones used in mild hyperthermia therapy, was chosen as the main component of the formulation. As tumor cells have a lower pH than non-tumor cells, the pH-sensitive lipid CHEMS (cholesteryl hemisuccinate) [41,42] was included in the nanosystems, with the purpose of attaining a pH-triggered release. Poly(ethylene glycol) (PEG) was also incorporated in the magnetoliposomes (through the PEGylated lipid DSPE-PEG) to ensure prolonged systemic circulation by avoiding clearance by the immune system.

Electrophoretic light scattering measurements (determination of zeta potential) were performed at neutral and acidic pH, respectively 7.4 and 5, to investigate the pH-sensitivity of the magnetic liposomal formulations containing the polymorphic molecule CHEMS. The results are displayed in Table 4. The zeta potential of DPPC magnetoliposomes (100%) at pH = 7.4 and pH = 5 is also shown, for comparison.

DPPC magnetoliposomes are very slightly negative, due to the surface charge of citrate-coated magnetic nanoparticles. A value of −25.5 mV was reported for the zeta potential of citrate-coated magnetite at pH = 6, decreasing with increasing pH [43]. At neutral pH, the presence of CHEMS in the magnetoliposomes formulations of DPPC:CHEMS and DPPC:CHEMS:DSPE-PEG reinforces the negative surface charge, as the succinate headgroup is deprotonated at neutral pH (pK~5.8 [14]). The more negative zeta-potential value of −26.7 ± 1.10 mV for the magnetoliposomes of DPPC:CHEMS indicates an outermost location of the CHEMS molecule in this formulation. At acidic pH, below its pK value, the protonation of CHEMS leads to a decrease in zeta potential to a very slight negative charge (near neutral) of −0.73 ± 0.9 mV and −2.55 ± 0.89 mV for DPPC:CHEMS and DPPC:CHEMS:DSPE-PEG, respectively (Table 4). Additionally, the protonation of CHEMS enhances the formation of the hexagonal phase (H_II_), conferring a more fusogenic character to the nanosystems at this pH. Hence, it is possible to conclude that the magnetoliposomes of DPPC:CHEMS and DPPC:CHEMS:DSPE-PEG are pH-sensitive, being suitable for pH-triggered release of encapsulated drugs in the acidic tumor microenvironment.

The interaction of the magnetic liposomes with small unilamellar vesicles (SUVs) of Egg-PC (L-α-lecithin from egg yolk), here employed as biomembrane models, was monitored by dynamic light scattering (DLS), investigating their fusion capabilities under neutral and acidic pH. For that, the hydrodynamic diameters of magnetoliposomes (MLs), SUVs and the mixture of both nanosystems (after stabilizing 10 min) were measured at pH = 7.4 (PBS buffer) and pH = 5 (acetate buffer). The outcomes of this experiment are shown in Table 5.

In general, the formation of generally monodisperse systems (PdI < 0.3) can be observed. The small hydrodynamic diameters of Egg-PC SUVs, at both pH 7.4 and 5, are in accordance with previous values reported for this type of vesicle [44]. At neutral pH, hydrodynamic sizes below or around 200 nm were measured, suitable for therapeutic applications considering that the enhanced permeation and retention (EPR) effect is guaranteed for nanocarriers’ sizes lower than 400 nm, while being more effective at diameters below 200 nm [45]. The larger negative surface charges (at pH = 7.4) of DPPC:CHEMS MLs (Table 4) contribute to a reduction in the mean size of this magnetic liposomal formulation, owing to a decrease in aggregation promoted by electrostatic repulsions.

An increase in size was observed for the mixture containing SUVs and MLs, supporting the fusion between the two types of nanostructures. As expected, in an acidic environment, the increase in size was even more pronounced, due to the lamellar-to-inverted hexagonal phase transition of CHEMS that supports membrane fusion at pH 5. The MLs of DPPC:CHEMS were the best candidates for pH-sensitive fusion with the largest size difference for the mixture of SUVs and MLs at pH 7.4 and 5. The lower increase obtained for the mixture of SUVs and MLs of DPPC:CHEMS:DSPE-PEG, at pH = 5, may be related to PEG molecules forming a hydrophilic corona that suppressed membrane fusion. In fact, PEGylation have shown poor endosomal escape through membrane fusion and lack of loaded molecules release in lysosomes [46]. However, recent studies have shown that the use of cleavable PEG derivatives, which are easy to break under pathological conditions, can facilitate the membrane fusion while keeping the extended drug circulation time [47].

**Figure 7:**
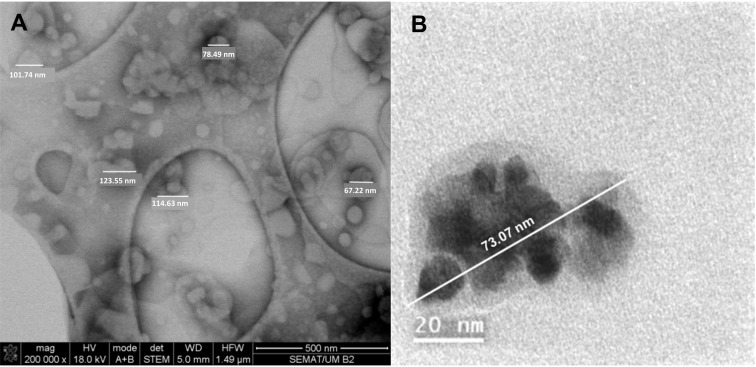
(**A**) SEM image of magnetoliposomes of DPPC (100%) based on citrate-stabilized nanoparticles, showing spherical structures around 100 nm size. (**B**) TEM image of a magnetoliposome.

Scanning electron microscopy (SEM) was used for the morphological assessment of the developed MLs. Despite not being ideal for analyzing liposomes, as it requires sample drying or fixing, SEM can provide general information on the concentric structure, as well as give details on size and spherical morphology [48]. An SEM image of the MLs based on citrate-stabilized nanoparticles is displayed in Figure 7A.

In general, single-layer magnetic liposomes with spherical shape and diameters around 100 nm were obtained. As expected, these results reveal slightly smaller diameters than the size distribution obtained from the DLS measurements, as the latter comprise the liquid layer around the nanosystem, while SEM measures the size of the dry nanoparticles. Additionally, aggregation in aqueous media can lead to larger average sizes. On the other hand, the fragmented and smaller size structures may result from the perturbation from the high-vacuum conditions required for sample preparation in SEM. A TEM image in Figure 7B illustrates a single magnetoliposome, evidencing the presence of magnetic nanoparticles with sizes smaller than 10 nm.

### 3.3. Drug-Loaded Magnetic Liposomes

#### 3.3.1. New Antitumor Thienopyridine Derivatives

As other thienopyridine derivatives previously synthesized and described [18,19,20,21,22], Compounds A and B are fluorescent in several solvents (except in water). This is an advantage for monitoring the incorporation of these compounds in the developed magnetic bionanosystems, as fluorescence provides a versatile method allowing for the determination of lower concentrations than UV-Visible absorption spectroscopy.

**Figure 8:**
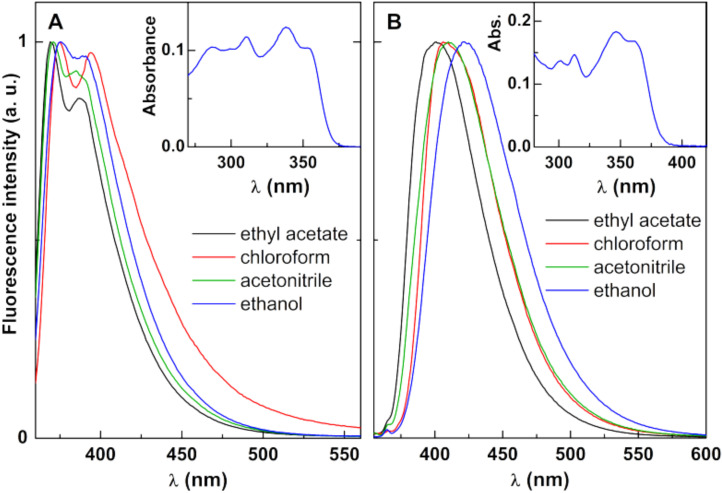
Normalized fluorescence spectra of 1 × 10−6 M solutions of Compounds (A) (λexc = 350 nm) and (B) (λexc = 330 nm) in different solvents. Insets: Absorption spectra in ethanol, as examples.

Considering these properties, UV-Vis absorption and fluorescence measurements were carried out for both compounds in different solvents and fluorescence quantum yields were estimated. The maximum absorption and emission wavelengths, molar absorption coefficients and fluorescence quantum yields are displayed in Table 6. Compound B is more fluorescent than Compound A, with quantum yields between 5% and 10%, while Compound A presents emissive quantum yields around 2%. Normalized fluorescence spectra are presented in Figure 8, with examples of absorption spectra as insets. A general red shift in polar and protic solvents (acetonitrile, ethanol) can be observed, together with a loss of vibrational structure and band enlargement, especially for Compound B, indicative of the intramolecular charge transfer character of the excited state [26]. This behavior points to a moderate sensitivity of the compounds’ emission to the environment.

The photophysical characterization of Compounds A and B (Figure 8 and Table 6) in several solvents is important to understand: not only are the compounds fluorescent in different environments, but their emission is also sensitive to the surrounding media. Understanding this will allow for determining compound encapsulation efficiencies and localization in magnetoliposomes—relevant parameters to be considered when assessing a nanocarrier performance.

#### 3.3.2. Magnetic Liposomes with Encapsulated Drugs

**Figure 9:**
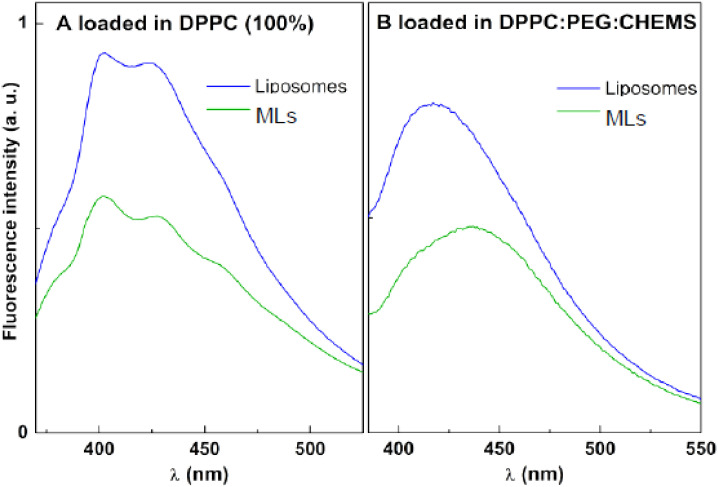
Fluorescence spectra of compounds loaded in liposomes and magnetic liposomes (**A**): Compound A loaded in DPPC (100%); (**B**): Compound B loaded in DPPC:PEG:CHEMS (80:20:0.4).

The antitumor Compounds A and B were loaded in magnetoliposomes by co-injection with the lipids the formulation. The encapsulation of Compounds A and B can be followed by fluorescence emission, taking into account the usual fluorescence quenching promoted by the magnetic nanoparticles. In Figure 9, a strong fluorescence inhibition is observed for both compounds when compared with the emission in neat liposomes (in the absence of the magnetic component). This behavior is common to all the lipid formulations studied and was already reported in previous works with other encapsulated drugs (including different thienopyridine derivatives) in magnetoliposomes [21,22,49].

Fluorescence anisotropy measurements are also useful in estimating the main location of the potential drugs in the nanocarriers (Table 7). In fact, fluorescence anisotropy, r, is related to the microviscosity, *η*, of the environment, through Equation (4) [26],

(4) $$\frac{1}{r}=\frac{1}{r_{0}}\left(1+\frac{\tau }{\tau_{c}}\right),$$

where *r*_0_ is the fundamental anisotropy, τ  is the excited-state lifetime of the fluorophore, and $\tau_{c}\;$ is the rotational correlation time, given by $\tau_{c}=\left(V_{h}\eta \right)/\left(k_{B}T\right)$, and with being V_h_ the hydrodynamic volume, *k*_B_ the Boltzmann’s constant and *T* the absolute temperature. The fundamental anisotropy can be estimated from the value in a very viscous solvent (e.g., glycerol).

In Table 7, a general decrease in anisotropy can be observed when the temperature is higher (55 °C) than that of the DPPC phase transition. This indicates that Compounds A and B detect the transition of the lipid bilayer (from gel to the liquid-crystalline phase) and, therefore, are mainly located in the membrane. An increase in the steady-state anisotropy values is predicted from the decrease in the excited-state lifetime (according to Equation (4)). When the temperature is raised from 25 °C to 55 °C, the excited-state lifetime decreases, owing to the enhancement of non-radiative deactivation pathways (especially the rate constant for internal conversion from the first singlet excited state to the ground state). However, a decrease in fluorescence anisotropy is observed, which can only be attributed to a decrease in the rotational correlation time of the fluorescent compound, which arises from the decrease in membrane microviscosity upon transition from the gel to the liquid-crystalline phase.

It should also be noticed that the compounds have very low anisotropy values in the DPPC (100%) formulation, and probably are in a hydrated and fluid environment in these nanostructures. On the other hand, CHEMS may facilitate compounds’ penetration in the membranes, by fluidizing the DPPC rigid phase, as happens with cholesterol [50]. This is reflected by the higher anisotropy values in formulations containing CHEMS.

The encapsulation efficiencies of both antitumor compounds in the several magnetoliposomes formulations were determined and the results are presented in Table 8.

The encapsulation efficiencies, above 88%, for both compounds are very reasonable and no significant difference between the several MLs formulation were observed. Only for Compound B was a slight increase in encapsulation efficiency obtained for PEGylated MLs, indicating that this formulation is able to better retain this drug. Hence, at neutral pH, the lipid formulations of magnetic liposomes do not sufficiently affect membrane fluidity to influence compound encapsulation.

#### 3.3.3. Förster Resonance Energy Transfer Assays

To further investigate whether the drug-loaded pH-sensitive MLs can deliver the encapsulated drug to model membranes (SUVs), Förster resonance energy transfer (FRET) assays were carried out using magnetoliposomes of DPPC:CHEMS and DPPC:CHEMS:DSPE-PEG loaded with Compound A. In these assays, this drug acts as the energy donor, while the hydrophobic dye curcumin in Egg-PC SUVs was used as energy acceptor. FRET occurs when a donor fluorophore in the excited state transfers its excitation energy to an acceptor moiety in the ground state through a non-radiative process. FRET between two fluorescent molecules is expected to be efficient if the donor–acceptor distance is below 100 Å [26].

Assuming that membrane fusion between MLs and SUVs promotes the approximation between Compound A (donor) and curcumin (acceptor) within this distance range, the fusogenic capability was assessed. The emission spectra of MLs loaded with Compound A and of the mixture containing both MLs loaded with Compound A and SUVs containing curcumin were measured, exciting only the donor at 350 nm. FRET efficiency, Φ_FRET_, representing the proportion of donor molecules that have transferred their excess energy to acceptor molecules, was calculated by taking the ratio of the donor-integrated fluorescence intensities in the presence of acceptor (F_DA_) and in the absence of acceptor (F_D_), through Equation (5). Here, the spectrum of MLs loaded with Compound A was used to measure the emission of the donor in the absence of an acceptor, while the spectrum of the mixture of loaded MLs and SUVs incorporating curcumin was used as the donor emission in the presence of an acceptor.

(5) $$\Phi_{\mathrm{FRET}}=1-\frac{F_{\mathrm{DA}}}{F_{D}}$$

In cells, the pH value drops from early endosomes (pH = 6.5) to late endosomes (pH = 6) and to lysosomes (pH = 4.5–5) [51]. The tumor microenvironment is also acidic. Considering the objective of obtaining pH-sensitive magnetoliposomes by the inclusion of CHEMS, together with temperature sensitivity promoted by DPPC, these assays were performed at pH = 7.4 (normal physiological pH) and 5 (pH of tumor environment), and at temperatures of 25 °C (below phase transition temperature of DPPC) and 45 °C (above DPPC transition and at mild hyperthermia condition). The results are summarized in Table 9.

At pH = 5, higher FRET efficiencies were obtained, indicating a higher fusion ability between the MLs and SUVs at acidic conditions. Cholesteryl hemisuccinate evidences the capability of adopting a lamellar structure upon hydration in alkaline or neutral media [15], promoting membrane fusion upon acidification, due to the preference of neutral form for the inverted hexagonal phase (H_II_) [14]. Additionally, a rise in temperature also increases the interaction between both nanosystems, with a larger membrane fusion and subsequent shorter distance between Compound A and curcumin (higher $\Phi_{\mathrm{FRET}}$). The lipid DPPC undergoes the gel-to-liquid crystalline phase transition at 41 °C; hence, at 45 °C, the increased membrane fluidity is expected to enhance membrane fusion. The DPPC:CHEMS:DSPE-PEG formulation has similar fusogenic sensitivity to the environment, considering the larger difference in FRET efficiencies between normal conditions (neutral pH and room temperature) and tumor environment under hyperthermia treatment (acidic pH and higher temperature). Therefore, the PEGylated nanocarrier maintains the pH and thermo-sensitive capabilities. Overall, the MLs based on citrate-stabilized Ca_0.5_Mn_0.5_Fe_2_O_4_ nanoparticles are very promising for antitumor drug delivery promoted by an external trigger in cancer therapy.

## 4. Conclusions

In this work, Ca_0.5_Mn_0.5_Fe_2_O_4_ nanoparticles were synthesized by co-precipitation (in the presence of citrate) and by the sol–gel technique. XRD measurements confirmed a pure crystalline phase of Ca_0.5_Mn_0.5_Fe_2_O_4_ NPs prepared by the first method. These nanoparticles showed a high saturation magnetization of 53.91 emu/g and superparamagnetic properties.

Magnetoliposomes of DPPC:CHEMS and DPPC:CHEMS:DSPE-PEG based on citrate-stabilized Ca_0.5_Mn_0.5_Fe_2_O_4_ nanoparticles, with sizes around 100 nm, were prepared. The presence of CHEMS in the liposomal formulation granted pH-sensitivity to the nanosystem, with a very slight negative charge in an acidic environment and a higher negative zeta-potential value at neutral pH. High encapsulation efficiencies, above 88%, were obtained for two new antitumor thienopyridine derivatives in the magnetic liposomes. FRET assays confirmed that the drug-loaded magnetoliposomes of DPPC:CHEMS and DPPC:CHEMS:DSPE-PEG display a higher fusogenic capability at acidic pH and high temperature. Hence, we developed magnetoliposomes suitable for temperature and pH-triggered release of anticancer drugs in the tumor microenvironment in combination with magnetic hyperthermia.

## Figures and Tables

**Table 1:** Growth inhibitory concentration values (GI_50_ ± SD; SD being the standard deviation) for Compounds A and B and DOX in tumor cells [16].

	HCT-15 (nM)	NCI-H460 (nM)
Compound A	5.6 ± 0.6	>75
Compound B	10.8 ± 1.1	17.0 ± 1.2
Doxorubicin	353.3 ± 24.2	25 ± 0.8

**Table 2:** Calculated R_P_ and χ^2^ parameters, phase sizes and percentages obtained by Rietveld refinement of X-ray diffraction patterns of Ca_0.5_Mn_0.5_Fe_2_O_4_ nanoparticles stabilized by citrate (A) or obtained through the sol–gel technique (B). *i* is the degree of inversion; $f_{Ca}^{T}$ is the fraction of Ca^2+^ in tetrahedral sites.

Sample	O_x,y,z_ (*)	*i*	$f_{Ca}^{T}$	Phase Size (nm)	Lattice Constant (nm)	Hematite (wt%)	R_P_	χ^2^
A	0.3816 0.3819 0.3795 0.3780	1 0.5 (+) 0.5 (+) 0 (+)	0 0 1 (+) 1 (+)	7.6 7.5 7.7 7.7	0.8427 0.8427 0.8430 0.8531	-	9.19 9.20 9.47 9.48	1.26 1.26 1.33 1.33
B	0.3848	1	0	10.2	0.8374	27.3	9.43	1.57

(*) Value of O_x,y,z_ in CIF file 2300618 is 0.25053 (+) fixed.

**Table 3:** Saturation magnetization (M_s_), remnant magnetization (M_r_), M_r_/M_s_ ratio and coercive field (C) for Ca_0.5_Mn_0.5_Fe_2_O_4_ nanoparticles, at room temperature.

	M_s_ (emu/g)	M_r_ (emu/g)	M_r_/M_s_	C (Oe)
Citrate-stabilized Ca_0.5_Mn_0.5_Fe_2_O_4_	53.91	0.95	0.02	13.90
Ca_0.5_Mn_0.5_Fe_2_O_4_ prepared by sol–gel	26.68	3.88	0.15	96.77

**Table 4:** Zeta potential values of the magnetic liposomes of several compositions, measured by electrophoretic light scattering.

Formulation	pH	Zeta Potential (mV)
DPPC (100%)	7.4	−1.83 ± 0.65
	5	−1.75 ± 0.82
DPPC:CHEMS (80:20)	7.4	−26.7 ± 1.10
	5	−0.73 ± 0.9
DPPC:CHEMS:DSPE-PEG (80:20:0.4)	7.4	−17.0 ± 0.9
	5	−2.55 ± 0.89

**Table 5:** Hydrodynamic diameter and polydispersity index (PdI) values of magnetic liposomes, SUVs and the mixture of both nanosystems, measured by dynamic light scattering (DLS).

Formulation	Hydrodynamic Size (nm)		PdI	
	pH = 7.4	pH = 5	pH = 7.4	pH = 5
SUVs	96.5 ± 2.4	92.3 ± 12	0.27 ± 0.01	0.27± 0.01
MLs (DPPC:CHEMS)	149.9 ± 17	203.6 ± 10	0.29 ± 0.01	0.27 ± 0.04
SUVs + MLs (DPPC:CHEMS)	171.6 ± 2.2	597.3 ± 58	0.25 ± 0.01	0.29 ± 0.04
MLs (DPPC:CHEMS:DSPE-PEG)	213.2 ± 1.1	225.6 ± 19	0.24 ± 0.001	0.28 ± 0.002
SUVs + MLs (DPPC:CHEMS:DSPE-PEG)	336.1 ± 121	376.1 ± 66	0.24 ± 0.027	0.25± 0.050

**Table 6:** Maximum absorption (λ_abs_) and emission (λ_em_) wavelengths, molar absorption coefficient values (ε) and fluorescence quantum yields (Φ_F_) calculated for Compounds A and B (*sh:* shoulder).

Solvent	λ_abs/_nm (ε/10^5^ M^−1^ cm^−1^)		λ_em_/nm		Φ_F_	
	Compound A	Compound B	Compound A	Compound B	Compound A	Compound B
Ethyl acetate	338 (1.37)	348 (2.07)	371; 387	401	0.016	0.05
Chloroform	338 (1.39)	352 (1.73)	375; 392	406	0.020	0.08
Acetonitrile	338 (1.27)	348 (1.79)	371; 385 (*sh*)	410	0.017	0.05
Ethanol	338 (1.24)	350 (1.76)	375; 389 (*sh*)	421	0.018	0.10

**Table 7:** Fluorescence anisotropy values of Compounds A and B in magnetic liposomes (MLs).

System	Formulation	Compound A	Compound B
MLs	DPPC	0.08 (25 °C) 0.06 (55 °C)	0.03 (25 °C) 0.02 (55 °C)
	DPPC:CHEMS	0.14 (25 °C) 0.10 (55 °C)	0.14 (25 °C) 0.11 (55 °C)
	DPPC:CHEMS: DSPE-PEG	0.11 (25 °C) 0.07 (55 °C)	0.14 (25 °C) 0.12 (55 °C)
Glycerol	-	0.33 (25 °C)	0.30 (25 °C)

**Table 8:** Values of encapsulation efficiencies (in percentage) for the studied antitumor compounds loaded in DPPC magnetoliposomes (standard deviation is from three independent assays).

Nanosystem	Formulation	Compound A	Compound B
MLs	DPPC	99.1 ± 0.2	89.0 ± 3.0
	DPPC:CHEMS	98.6 ± 0.1	88.4 ± 2.8
	DPPC:CHEMS:DSPE-PEG	98.2 ± 0.2	91.2 ± 0.3

**Table 9:** Values of energy transfer efficiencies ($\Phi_{\mathrm{FRET}})$, in percentage, at different pH and temperature conditions.

Formulation	pH	Temperature (°C)	$\Phi_{\mathrm{FRET}}\;(\%)$
DPPC:CHEMS	7.4	25	10.9
		45	22.5
	5	25	23.5
		45	26.6
DPPC:CHEMS:DSPE-PEG	7.4	25	18.4
		45	26.9
	5	25	28.9
		45	35.3

## Data Availability

Not applicable.
